# Indirect optimization of staphylokinase expression level in dicistronic auto-inducible system

**DOI:** 10.1186/s13568-022-01464-0

**Published:** 2022-09-22

**Authors:** Fatemeh Sadat Shariati, Malihe Keramati, Reza Ahangari Cohan

**Affiliations:** Department of Nanobiotechnology, New Technologies Research Group, Pasteur Institute of Iran, Tehran, Iran

**Keywords:** Expression, Optimization, *Escherichia coli*, Response surface methodology, Green fluorescent protein, Design of experiment

## Abstract

Design of experiment (DOE) is a statistical approach for designing, performing, and interpreting a large set of data with the minimum number of tests. In our previous study, we developed a novel Hsp27 SILEX system for production of recombinant proteins. In the present study, we optimized indirectly the most effective factors including inoculation load, self-induction temperature, and culture media on autoinduction of staphylokinase (SAK) expression using RSM methodology and fluorometry. The expression level of SAK was assayed at different runs after 6 h incubation at 90 rpm. The results indicated all parameters significantly affect the SAK expression level (p < 0.05). The optimum expression condition was obtained with an inoculation load of 0.05, a temperature of 25 °C, and TB culture medium. The analysis of variance with a R^2^ value of 0.91 showed that a quadratic model well described this prediction (p < 0.05). Applying the optimized condition led to an approximately fourfold increase in the SAK expression level (from 1.3 to 5.2 µg/ml). Moreover, the recombinant protein was purified using immobilized metal affinity chromatography and the activity was also confirmed by semi-quantitative caseinolytic method.

**Supplementary Information:**

The online version contains supplementary material available at 10.1186/s13568-022-01464-0.

## Introduction

Identification of high producer colons is a milestone for production of high amount of recombinant proteins at a reasonable cost. Therefore, rapid screening and expression optimization at the shortest time with the lowest cost are essential in the production of recombinant proteins, especially at an industrial scale (Hertzberg et al. 2000; Stephens et al. 2002; Vincentelli et al. 2011).

Among prokaryotic hosts, *Escherichia coli* (*E. coli*)-based expression systems have gained the most interest for the production of recombinant non-glycosylated proteins due to their high expression level, simplicity, and low cost (Akbarzadeh et al. 2015; Huang et al. 2012). However, measurement of the expression using routine or traditional techniques including SDS-PAGE, immunoblot analysis, and biological activity assays is time-consuming and labor-intensive (Hui et al. 2018). In a study by Huietal et al*.*, an indirect expression of target protein in *E. coli* was monitored based on a mCherry reporter. The reporter gene was inserted in the downstream of target protein ORF (Open Reading Frame). Therefore, the expression of the target gene was positively correlated to the expression of mCherry reporter. The method relied on an indirect and quantitative determination of recombinant expression level by measuring the fluorescent intensity of mCherry during the fermentation (Hui et al. 2018). In another approach, a microfluidics platform was used to optimize indirectly the protein expressions in the cells encapsulated with the biocompatible gel beads. In this platform, the encapsulated cells (bacteria and yeast) were fluorescently labeled to monitor and isolate the high-expressing clones using flow cytometry (Napiorkowska et al. 2021).

Recently, we developed a dicistronic autoinducible expression system, called SILEX (Self Inducible Expression System), to facilitate the screening of high-expressing clones using fluorometry. This autoinducible system was first introduced in 2016 and has advantages over other previous self-inducible systems (Briand et al. 2016). In our system, the model protein genes including staphylokinase (SAK), interleukin-2, and romiplostim were cloned at the first position and the sequence encoding enhanced Green Fluorescent Protein (eGFP) was inserted at the second position (Shariati Norouzian et al. 2021). The expression of model proteins was performed using T7 promoter under the control of the *lac* operon and Hsp27 as an autoinducer (Kuhlman et al. 2007; Müller-Hill 2011). Our prior results revealed the amount of expression at two positions is proportional to each other. Therefore, it can be concluded that optimization of eGFP expression at the second position can be used for the optimization of expression at the first position.

The expression of recombinant proteins in prokaryotic systems can be influenced by multiple parameters including the type of host and vector, codon optimization, co-expression of cellular chaperones, engineering of transcription and translation regulatory elements, deletion of proteases, signal peptide engineering, the addition of solubility-enhancing tags, the composition of culture medium, and optimization of the growth condition such as temperature, inducer concentration, inoculation load, post-induction period, and pH (Akbarzadeh et al. 2015; Briand et al. 2016). Optimization of expression is often carried out by varying these parameters at the same time and modeling the variables and their interactions on a response using statistical and mathematical approaches (Montgomery. 2017; Papaneophytou. 2019). Response surface methodology (RSM) is a statistical method that is usually utilized for designing, conducting, analyzing, and interpreting experiments. RSM has several advantages over traditional one-factor-at-a-time (OFAT) approach such as determination of the interactions among different factors in a process, identification the optimum values within fewer experimental runs, and cost reduction (Akbarzadeh et al. 2015).

In this study, central composite design (CCD) was used for indirect optimization of SAK expression in Hsp27 SILEX system at 5-levels using three factors (temperature, inoculation load, and the culture medium) in 96-well microplates (Sarduy et al. 2012). This approach helps us reach to an optimum expression condition with a small run of experiments in comparison to a full factorial design (Akbarzadeh et al. 2015; Rudakiya. 2019). Finally, the overexpressed recombinant SAK protein was purified for subsequent bioactivity measurement.

## Methods and materials

### Bacterial strain and culture media

BL21 (DE3) *Escherichia coli* strain carrying the recombinant plasmids (pET28a-*sak rbs egfp* and pET21-*hsp27*) were constructed in our previous study (Shariati Norouzian et al. 2021). Three different media supplemented with ampicillin (100 µg/ml) and kanamycin (35 µg/ml) were used for the optimization including LB [10 g/L tryptone, 5 g/L yeast extract and 10 g/L sodium chloride], TB [12 g/L tryptone, 24 g/L yeast extract, 12.54 g/L K2HPO4, 2.31 g/L KH2PO4,and 5 g/L glycerol dissolved in deionized water], and 2YT medium [16 g/L tryptone, 10 g/L Bacto yeast extract, 5 g/L NaCl dissolved in deionized water] for bacterial culture. All ingredients for medium preparations were purchased from Sigma, USA.

### Experimental design and cultural conditions

In general, 39 experiments were designed using Design-Expert software version 11.0.3. The SAK expression (as a response) was indirectly measured by the intensity of fluorescent signals of eGFP (Additional file 1: Table S1). The experiments were performed according to Table 1 with five replicated center points. A three-variable CCD at five levels was used to optimize the protein expression (Table 2). A single clone was inoculated into 5 ml of LB, TB, and 2YT medium supplemented with the appropriate antibiotics and incubated for 16 h at 37 ºC/170 rpm. After reaching an optical density (OD_600nm_) of 1.6, different inoculum loads (Ratio of the overnight culture to the culture media: 0.027, 0.03, 0.04, 0.05, 0.053) were added according to the designed experiments. Then, 10 µl of seed cultures were added to 190 µl of culture media in 96-well microplates. The microplates were then incubated at different temperatures (23, 25, 31, 37, and 39 °C) at 90 rpm (Table 1). The fermentations were terminated after 6 h of cultivation and the intensity of fluorescence signals was measured using fluorometry (485 nm for excitation and 528 nm for emission, Epoch, BioTek, USA) in triplicate (Shariati Keramati et al. 2021). The SAK expression level was investigated according to measuring the fluorescent intensity and eGFP concentration vs fluorescent signals plot.

**Table 1:** The designed experiments for indirect optimization of SAK expression as a response

Run	Temperature (°C)	Inoculum load	Culture medium	Experimental eGFP Expression level (µg/ml)
1	23	0.040	TB	4.9
2	23	0.040	LB	3.8
3	37	0.050	LB	1.3
4	39	0.040	2YT	3.5
5	25	0.050	TB	5.2
6	25	0.050	2YT	4.0
7	37	0.030	LB	1.7
8	31	0.040	2YT	2.9
9	25	0.050	LB	3.8
10	39	0.040	LB	2.3
11	31	0.040	TB	2.7
12	37	0.050	2YT	2.3
13	25	0.030	2YT	2.8
14	31	0.040	2YT	3.5
15	31	0.027	2YT	2.3
16	25	0.030	LB	2.9
17	31	0.040	2YT	2.6
18	31	0.040	LB	2
19	31	0.040	TB	3.3
20	31	0.040	TB	3.3
21	39	0.040	TB	2.8
22	25	0.030	TB	3.2
23	37	0.030	2YT	2.6
24	31	0.040	2YT	3.3
25	31	0.040	LB	2.1
26	31	0.040	LB	2.0
27	23	0.040	2YT	4.3
28	31	0.040	LB	2.1
29	31	0.040	TB	3.3
30	31	0.027	LB	1.6
31	31	0.027	TB	1.9
32	31	0.040	LB	2.8
33	37	0.050	TB	2.3
34	31	0.040	TB	3.5
35	37	0.030	TB	1.6
36	31	0.040	2YT	3.2
37	31	0.053	TB	2.6
38	31	0.053	LB	2.1
39	31	0.053	2YT	2.3

**Table 2:** The variables and their levels used in central composite design

Variable	Levels				
	− α	− 1	0	1	+ α
Self-induction Temperature	23	25	31	37	39
Inoculation Load	0.027	0.03	0.04	0.05	0053
Media	LB	TB	2YT	3 Levels	

### Expression analysis and protein purification

The SAK and eGFP expressions were analyzed by SDS-PAGE and Coomassie blue staining using a Bio-Rad electrophoresis system (USA). Briefly, a single clone was inoculated into 5 mL of TB medium and incubated at 37ºC/170 rpm for 16 h. Then, 100 µl overnight bacterial culture (at inoculation load of 0.05_)_ was added to a 5 ml TB medium and incubated for another 16 h at 25ºC/250 rpm. Two milliliters of culture medium containing the expressed SAK were collected and centrifuged for 10 min at 7000 g. Then, the pellet was resuspended in a mixture of 5 × sample loading (100 µl) containing 0.004% bromophenol blue, 2-mercaptoethanol (5%), 50 mM phosphate buffer at pH 7.0, and 10% SDS and heated for 10 min at 100 °C and eventually analyzed by SDS-PAGE. Following the expression, the recombinant SAK was purified via affinity chromatography. Briefly, the pellet was resuspended in a lysis buffer containing 50 mM NaH_2_PO_4_.2H_2_O, 300 mM NaCl, and 1 mg/ml lysozyme adjusted to pH 8 and lysed by sonication on ice for 20 min. The lysate was centrifuged for 25 min at 14,000 g/ 4 °C and the supernatant was loaded on an equilibrated Ni–NTA resin (Qiagen, USA) for 1 h at 4 °C according to the manufacturer's protocol. The resin was washed several times with the wash buffer (300 mM NaCl, 20 mM imidazole, 50 mM NaH_2_PO_4_.2H_2_O, pH 8). After that, the SAK protein was eluted using an elution buffer contain 250 mM imidazole.

### SAK activity measurement

The activity of the purified SAK at optimized conditions was assayed by semi-quantitative radial caseinolytic method on 5% skim milk agar plates (0.05 g/mL skim milk, 0.04 g/mL LB agar, 50 mM Tris–HCl, 0.15 M NaCl, pH 7.5) (Shariati Norouzian et al. 2021). Briefly, a clone of dicistronic Hsp27 SILEX system was inoculated into a 5 mL TB medium (with 100 μg/mL ampicillin and 35 µg/mL kanamycin) and incubated in a shaker incubator at 37ºC/170 rpm, overnight. Then, a 5 mL TB medium was inoculated with 100 µL of seed culture and incubated for 16 h at 37 ºC/250 rpm. The culture was centrifuged at 4000 g/ 4ºC and the pellet was resuspended in the lysis buffer (50 mM NaH_2_PO_4_.2H_2_O, 300 mM NaCl, and 1 mg/ml lysozyme, pH 7.5). The lysate and 8 µl plasminogen (142.85 µM) were poured into the holes and incubated at 37 ºC for 18 h. The SAK activity was measured for three different clones at the optimized conditions using Image J software (https://imagej.nih.gov/ij/index.html). The standard SAK (Prospect, 50,000 IU/mg) with and without plasminogen were used as positive and negative controls, respectively (Shagufta Naseer et al. 2014). Also, a clone from dicistronic Hsp27SILEX system was selected and the activity of SAK at optimized conditions was measured in triplicate using chromogenic assay and the data was compared with the standard.

### Statistical analysis

The models for optimization of SAK expression in SILEX system were generated by design expert software. The SAK expression levels were calculated using the second-order polynomial equation as a response. The effect of independent parameters on the response was investigated using variance analysis (ANOVA) and a p-value of less than 0.05 was considered significant. F-test was used to evaluate the significance of the model equation and terms. Multiple correlation coefficient (R^2^), adjusted R^2^, predicted R^2^, and adequate precision were used to assess the quality of model fitness. Also, the interactions and relationships between the variables and the response were portrayed through three-dimensional and contour surface plots.

## Results

### Optimization of SAK expression by statistical experimental design

In this research, CCD was utilized for modeling SAK expression in Hsp27 SILEX system. This model can be used for the prediction of parameters even at their borderline regions. The SAK and eGFP expressions were optimized according to influential factors (independent) including temperature, inoculation load, and culture medium by measuring the fluorescent signals. The relationship between the independent variables and the target response was explained by a second-order polynomial equation (Muntari et al. 2012). The modified second-order polynomial equation in the terms of coded factors was obtained by applying multiple regression analysis to the experimental data and explained the SAK expression as follows:

**SAK Expression** = 2.58—0.7255 **A** + 0.2559 **B**—0.4913 **C** [1] + 0.2882 **C** [2]—0.3534 **AB**—0.0466 **AC** [1]—0.2760 **AC** [2]—0.1059 **BC** [1] + 0.2330 **BC** [2] + 0.4455 **A**^**2**^—0.4595 **B**.^**2**^

Where, Y is the predicted independent variable (SAK expression) and A (Temperature), B (Inoculation), and C (Culture medium) are the independent variables.

Table 3 shows the ANOVA analysis for the modified model describing the SAK expression by the variables in Hsp27 SILEX system. The results of ANOVA analysis showed that the model is significant (p-value < 0.0001) and the possibility that the F-value of the model occurs due to the noise is less than 0.01%. The F-value (F: 28.17) of the model was measured by dividing the mean square of the model by the mean square of the residuals. The model was significant at *p* < 0.0001, or a level greater than 95%. Additionally, the F-value of lack of fit was measured by dividing the mean square of lack of fit by the mean square of pure error (F: 0.8075) and was less than the critical value of F (28.17). The lack of fit is a calculation of the failure of the model to fit the empirical data and the model is removed if the lack of fit is significant. The small F-value of lack of fit and also a p-value larger than 0.05 indicated that the lack of fit was not significant relative to the pure error and the model fitted the data properly (Guo et al. 2018).

**Table 3:** ANOVA analysis of SAK expression in dicistronic Hsp27 SILEX system by the second order polynomial model

Source	Sum of squares	df	Mean square	F-value	P-value	Significant
Model	27.30	11	2.48	28.17	< 0.0001	*
A-Temperature	11.39	1	11.39	129.25	< 0.0001	*
B-Inoculation	1.42	1	1.42	16.08	0.0004	*
C-Media	4.75	2	2.38	26.99	< 0.0001	*
AB	1.50	1	1.50	17.02	0.0003	*
AC	1.31	2	0.6575	7.46	0.0026	*
BC	0.5888	2	0.2944	3.34	0.0505	
A2	3.07	1	3.07	34.84	< 0.0001	*
B2	3.27	1	3.27	37.07	< 0.0001	*
Residual	2.38	27	0.0881			
Lack of Fit	1.19	15	0.0797	0.8075	0.6572	
Pure Error	1.18	12	0.0986			
Cor Total	29.67	38				

**Fig. 1:**
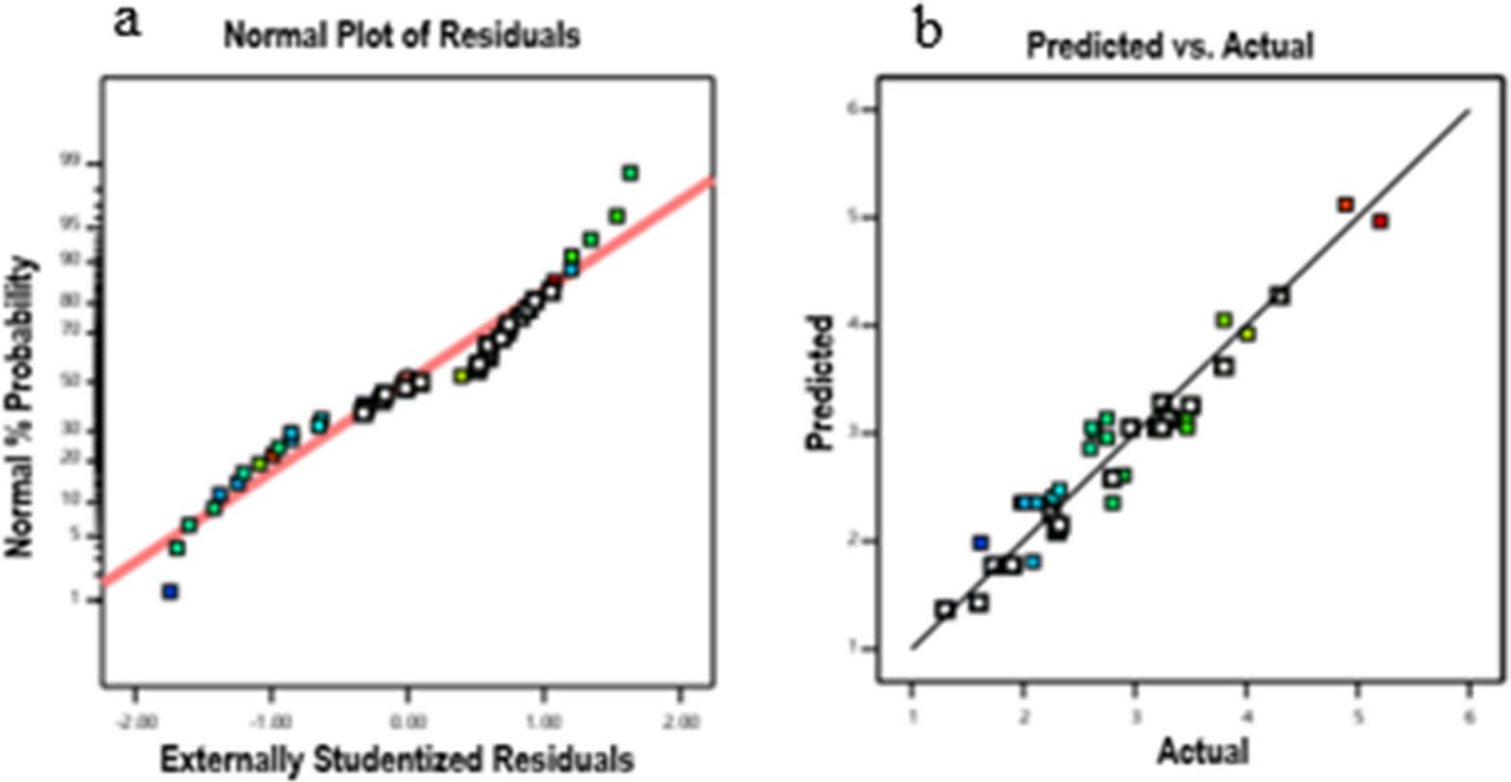
Normal probability and the relationship between the predicted and actual values graphs. (**a**) The plot showed that the data were normally distributed. (**b**) The plots showed the relationship between the predicted and actual values of SAK expression as a response at Hsp27 SILEX system. The closeness of scattered data to the diagonal line confirmed that model fitted the empirical data adequately

**Fig. 2:**
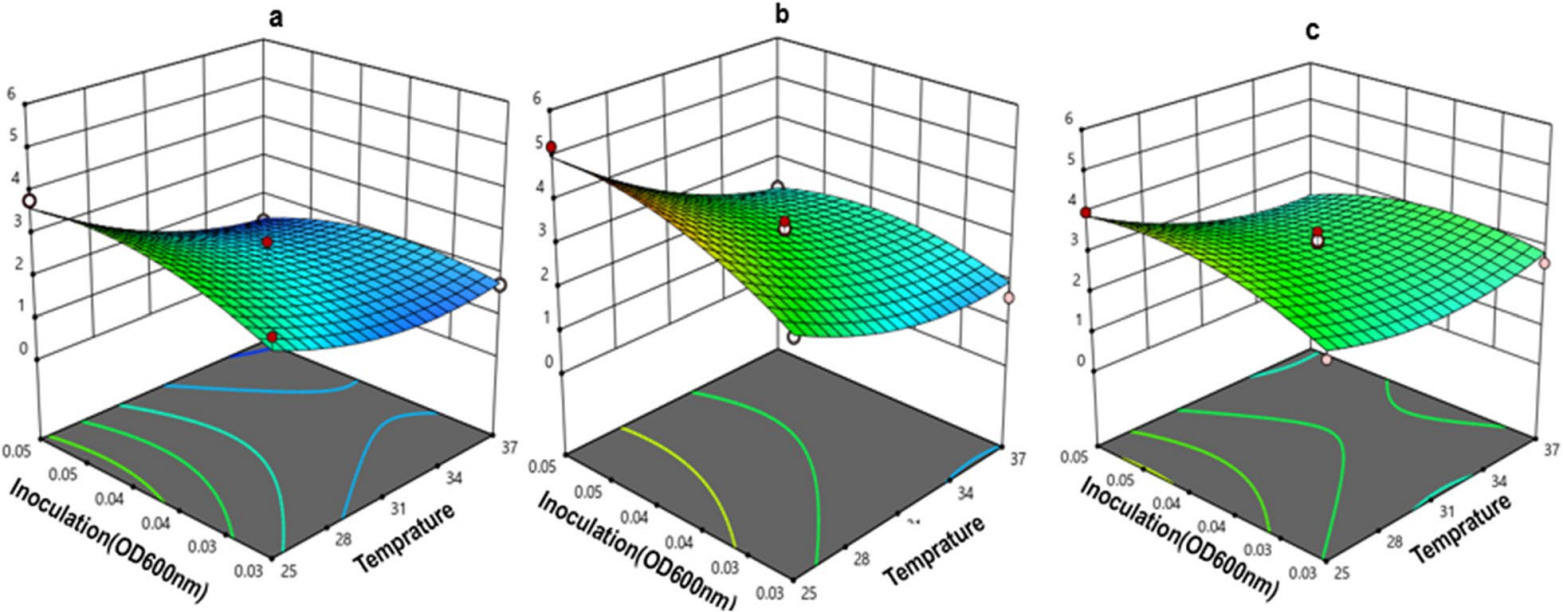
The effect of inoculum load and temperature on SAK expression at (**a**) LB medium, (**b**) TB medium, and (**c**) 2YT media

The predicted R^2^ value (0.84) was in reasonable agreement with the value of adjusted R^2^ (0.89) and the difference was less than 0.2. Adequate precision shows the signal-to-noise ratio and the high value of adequate precision confirmed the adequacy of model (values more than four are desirable). The adequacy of the model was further investigated by drawing the residual normal probability plot for finding the outlier data and limitations associated with the analysis. The result showed an approximately linear pattern that errors are distributed normally, whereas a non-linear pattern showed non-normality (Fig. 1). The curvature contour lines (three-dimensional response surface plots) indicated that the interactions of factors were significant. The effect of temperature-inoculum load interaction on SAK expression was shown in different culture media (Fig. 2). In this study, enhancing SAK expression with the same importance priority, and the boundaries of independent variables were selected in the range of − α and + α. The predicted optimal criteria were achieved as a temperature of 25 °C, an inoculation load of 0.05, and TB which resulted in a maximum SAK expression (5.2 µg/ml) (Table1).

### Expression analysis of SAK and eGFP by SDS-PAGE

**Fig. 3:**
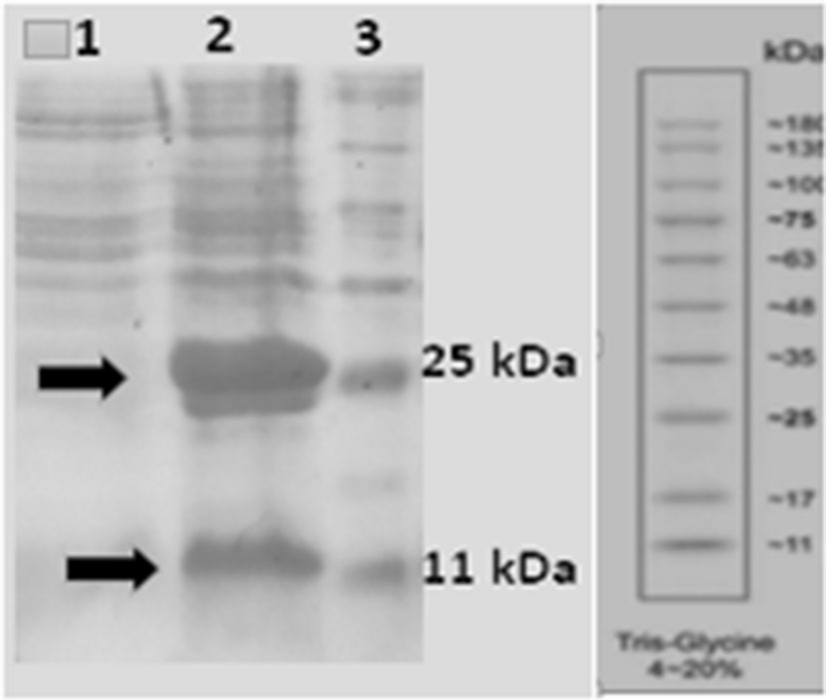
SDS-PAGE analysis of SAK and eGFP expressions in dicistronic Hsp27 SILEX system. Lane 1: 2 h after inoculation, Lanes 2: 16 h after inoculation, and Lane 3: Protein marker. The arrows indicate SAK and eGFP proteins at molecular weights of 15.5 and ~ 28 kDa, respectively. The protein marker molecular weights are 180, 135, 100, 75, 63, 48, 35, 25, 17, and 11 kDa

The expressions of SAK and eGFP at optimized conditions were assayed using 15% SDS-PAGE. The related bonds were seen at molecular weights of 15.5 kDa and ~ 28 kDa for SAK and eGFP, respectively (Fig. 3).

### Enzyme activity of SAK

**Fig. 4:**
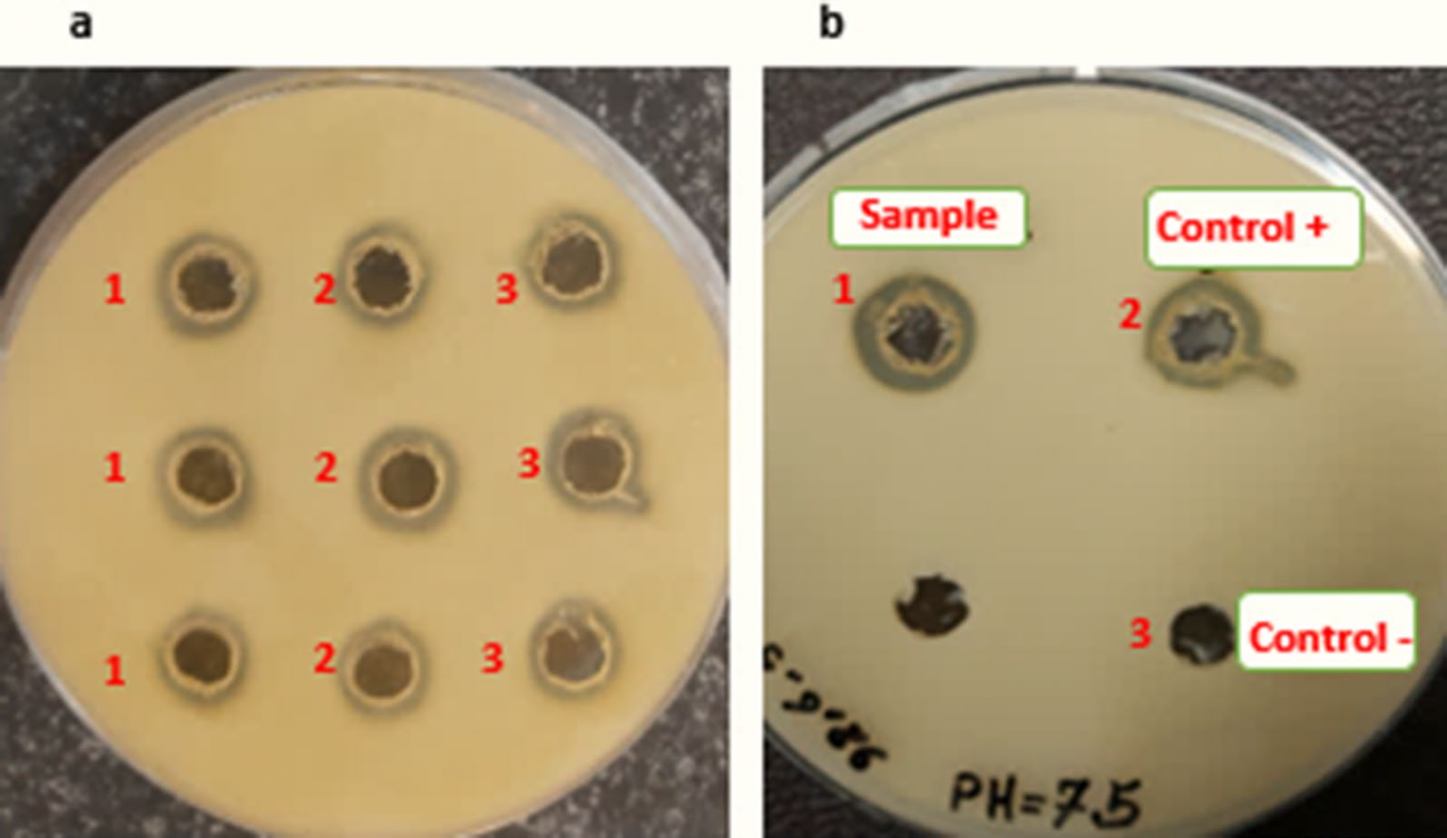
Radial caseinolytic assay for SAK activity measurement on 5% skim milk-agar plate. **a** shows the expression of SAK in the dicistronic SILEX system at optimized conditions for three clones (wells 1, 2, and 3 are replicates). **b** shows the positive and negative control for SAK activity [well 2: commercial SAK with plasminogen (As the positive control), and well 3: Commercial SAK without plasminogen (As the negative control)]

The biological activity of SAK following protein purification was assessed in triplicate by radial caseinolytic method for three different clones at optimized condition. As shown in Fig. 4, the diameter of clear zones around the wells indicating the proteolytic activity of the SAK (Table 4). The result showed that the purified SAK activity was ~ 289.55 ± 0.41 IU/mg after 6 h incubation at 25 °C/90 rpm in TB medium. While the mean of SAK activity at other conditions (e.g. in LB culture medium at 37 °C with an inoculation load of 0.05) was 143.59 ± 5.11 IU/mg.

**Table 4:** The results of radial caseinolytic assay for SAK activity

Sample	Clone 1	Clone 2	Clone 3	Positive control ^b^	Negative control ^c^
Clear zone (cm)	1.4700 ± 0.0081	1.4833 ± 0.0124	1.4766 ± 0.0047	1.5 ± 0.0081	0.61 ± 0.0081
RSD ^a^ (%)	0.5554	0.8408	0.3192	0.5443	1.33

Data are represented as Mean ± SD from three independent experiments

^a^Relative Standard Deviation

^b^Recombinant SAK with plasminogen

^c^Recombinant SAK without plasminogen

## Discussion

In this study, a dicistronic SILEX system was used for indirect and quantitative optimization of SAK expression at different conditions by measuring the fluorescent signals. Our developed system can get a lot of interest for facilitated screening and optimization of recombinant proteins at low cost and in the shortest time (Shariati Norouzian et al. 2021). The selection of experimental design in this system is performed based on the number and the type (categorical or continuous) of factors used for the optimization of SAK expression. RSM is selected as a scientific and effective method to improve the cultivation condition which allows evaluating the effects of the variables on expression level at dicistronic SILEX system. We tried to reach the maximum level of SAK expression in SILEX system by optimization of three effective parameters including inoculum load, self-induction temperature, and the culture medium. These variables were selected based on the related studies on the SILEX systems (Shariati Keramati et al. 2021; Shariati Norouzian et al. 2021). The adequacy of the predicted model was investigated by analysis of variance and the model terms with p-values larger than 0.05 were omitted from the model (Khodadadi et al. 2014). The results of experiments with a F-value of 28.17 and a p-value less than 0.0001 confirmed the significance of model. Moreover, the lack of fit that was not significant, clearly expresses that the obtained experimental responses (SAK expression) in this study properly fitted with the model. The highest amount of expressed SAK was obtained with in run 5 (5.2 mg/ml) while the lowest value was observed in run 3 (1.3 mg/ml). The results were analyzed by response surface methodology and a second-order polynomial equation was expressed as below (Koivunen et al. 2005).

$$y=\alpha 0+\sum_{i=1}^{n}\alpha iXi+\sum_{i=1}^{n}\alpha iiXi2+\sum_{i=1}^{n}\sum_{j=i+1}^{n}\alpha ijXiXj$$

The negative contribution of the temperature on the SAK expression level in the SILEX system can be attributed to more bacterial cell growth due to an increased in the temperature, which reduces the soluble protein expression during the induction phase and resulted in inclusion body formation (MacDonald et al. 2003). The results from the aforementioned studies by Muntari are in good accordance with our findings, and the maximum expression of recombinant proteins is highly favored by lower growth temperature (Schein et al. 1988). In another study by Vera et al*.*, a large number of recombinant proteins precipitated with increasing in the temperature from 15 to 37 °C. This is because the bacterial expression system provides sufficient time for the newly transcribed recombinant proteins to fold properly at a lower temperature (Vaz et al. 2015; Vera et al. 2007). On the other hand, lower temperature could improve the plasmid stability and the overall production yield of soluble protein (Hong et al. 2002). This fact was established that a temperature of 25 °C could significantly enhance the expression of the recombinant protein in comparison to the ordinary *E.coli* growth temperature at 37 °C.

Moreover, a maximum SAK expression level was achieved at an inoculum load of 0.05. The results showed that a higher inoculation load used under the optimized conditions has a greater function for increasing the expression. In fact, it is inferred that higher cell density at the inoculation point causes overproduction of a recombinant protein that leads to the production of heat shock proteins and subsequently higher SAK expression (Sarduy et al. 2012). The appropriate growth phase at the time of self-induction can affect protein expression. Also, for recombinant protein production, the composition of the medium must be carefully formulated and monitored, because it may have significant metabolic effects on both the cell growth rate and protein expression level (Sarduy et al. 2012). The result of our study showed that the maximum SAK expression level in the SILEX system was obtained in TB medium compared to LB medium which corresponds to Collins's study for evaluation of culture media on CHO (Chinese Hamster Ovary) expression (Collins et al. 2013). Also, our findings are in agreement with the result of a previous study by Vaz and et al*.*, that the higher cell density gained in TB medium can be explained by the larger number of nutritious components, such as amino acids, vitamin precursors, and glycerol as an energy and carbon source (Vaz et al. 2015). TB medium due to its high yeast extract content can provide a large amount of organic and inorganic nutrients and nitrogen for the bacteria thus favoring cell growth (Vaz et al. 2015). Furthermore, the culture medium composition can affect the yield of the recombinant protein expression by altering the bacterial host metabolism (Sivashanmugam et al. 2009). Our results demonstrated that the TB medium was a suitable medium for the production of the target protein. A high concentration of yeast extract, superior buffering capacity, and glycerol as the carbon source supplement lead to high biomass accumulation and high recombinant protein production (Collins et al. 2013).

## Supplementary Information

**Additional file 1: Table S1. **The fluorescent intensity measured in each run for indirect optimization of SAK expression.

## Data Availability

All data generated or analyzed during this study are included in the published article and are available from the corresponding author on reasonable request.

## Abbreviations

eGFP: Enhanced green fluorescent protein
SAK: Staphylokinase
LOD: Limit of detection
SILEX: Self-induced expression system
RFU: Relative fluorescent unit
RSM: Response surface methodology
ORF: Open reading frame
RSM: Response surface methology
OFAT: One-factor-at-a-time
CCD: Central composite design
RSD: Relative standard deviation
LB: Luria–Bertani
TB: Terrific Broth
CHO: Chinese Hamster Ovary
