# Supplementary material for: Indirect optimization of staphylokinase expression level in dicistronic auto-inducible system
Source: AMB Express. 2022 Sep 22;12:124. doi: 10.1186/s13568-022-01464-0 (PMC9500143; doi:10.1186/s13568-022-01464-0)
Supplement: Supplementary file 1 — Additional file 1: Table S1. The fluorescent intensity measured in each run for indirect optimization of SAK expression. [file 13568_2022_1464_MOESM1_ESM.docx]

**Table S1.** The fluorescent intensity measured in each run for indirect optimization of SAK expression.

| Run | Fluorescent Intensity (RFU) | Run | Fluorescent Intensity (RFU) |
| --- | --- | --- | --- |
| 1 | 22128.77 | **21** | 9813.85 |
| 2 | 15678.1 | **22** | 12159.56 |
| 3 | 1017.49 | **23** | 8641.01 |
| 4 | 13918.83 | **24** | 12745.98 |
| 5 | 23888.05 | **25** | 5708.88 |
| 6 | 16850.95 | **26** | 5122.46 |
| 7 | 3363.18 | **27** | 18610.23 |
| 8 | 10400.28 | **28** | 5708.88 |
| 9 | 15678.10 | **29** | 12745.98 |
| 10 | 6881.73 | **30** | 2776.76 |
| 11 | 9227.43 | **31** | 4536.03 |
| 12 | 6881.73 | **32** | 9813.85 |
| 13 | 9813.85 | **33** | 6881.73 |
| 14 | 13918.83 | **34** | 13918.83 |
| 15 | 6881.73 | **35** | 2776.76 |
| 16 | 10400.28 | **36** | 12159.56 |
| 17 | 8641.01 | **37** | 8641.01 |
| 18 | 5122.46 | **38** | 5708.88 |
| 19 | 12745.98 | **39** | 6881.73 |
| 20 | 12745.98 |  |  |
